# A place to start?

**DOI:** 10.1177/0308518X231198008

**Published:** 2023-09-23

**Authors:** Jamie Peck

**Affiliations:** The University of British Columbia, Vancouver, BC, Canada

**Keywords:** Economic geography, methodology, conjunctural analysis, uneven development, recombination

## Abstract

How do economic geographers determine where to begin their research projects, where to locate and delimit their case studies, where and how to “cut in” to problems? In the absence of self-evident or pregiven answers to these questions, the problem-cum-choice of where and how to start is inescapably tangled up with issues of preliminary conceptualization and indeed theorization, since cases are not so much found as made, being in various ways coproduced with different “theory-method packages.” There is (and can be) no singular or universal answer to these questions. Instead, this brief intervention outlines one rationale for getting “started,” founded as such rationales should be with reference a particular approach or mode of theorization. The approach here centers on the problematic of recombinant development, on the role of extended case-study designs, and on the still sparsely realized potential of conjunctural modes of analysis.

Where to begin? The question is a seemingly banal if also rather consequential one—certainly when it comes to the initiation of research projects the field of economic geography, where geographical discontinuity, localization, and uneven spatial development are elemental concerns. Of course, there are pragmatic, resourcing, and logistical issues (indeed constraints) to consider when thinking about where to begin and how to select a research site, but there is also a plethora of wider (and deeper) questions concerning research design, methodology, conceptualization, positionality, and theorization that cannot be avoided or even postponed. More to the point, these questions *should not* be avoided or postponed. But having registered where this intervention is going, it is appropriate to begin from where we are. Economic geographers, for all their other differences, tend to take seriously considerations of place, positionality, and perspective. “Context,” situation, embeddedness, and (relative) location are understood to matter; *where* economies take place, in an unevenly developed world, matters. Economic geographers, colleagues from other disciplines will sometimes point out, habitually feature the names of places in the titles of their papers and books, signaling the fact that these are concrete contexts, institutional settings, and social situations that *count*, conditioning both explanatory strategies and production of (theory) claims. It follows that decisions about where to start—what point of entry, where to cut in, how to engage—are not trivial, arbitrary, or matters of mere convenience. Methodologically speaking, these are decisions with consequences, and yet oddly enough there seems to be little explicit discussion of them.

Where economic geographers choose to start, how they situate and frame their interventions, and where they eventually go with their explanations, are certainly not self-evident or pregiven. They are choices, albeit conditioned and constrained choices. This translates into an open, exploratory, and creative mandate, which means that things rarely get stale or boring, even if the resulting proclivity to pursue real-time change and to keep on turning is not always compatible with more “systematic,” sustained or cumulative inquiries. Resistant to being hemmed in or pinned down, economic geography’s generally forward-facing methodological culture is inclined toward improvisation, eclecticism, and repeatedly widening the circle of a restlessly critical heterodoxy, rather than deferring to prescribed routines or established orthodoxies; theoretical frameworks are conventionally understood to be plastic and pliable, rather than rigorously predetermined; theorizing mostly occurs in dialog with empirical investigation, a measure of inductive openness being favored over deductive discipline and (potential) foreclosure with the goal of explanatory closure; and there is not much tolerance for boundary policing or sticking to an established lane. This is not to say that research design, site selection, and case-study specification are determined willy-nilly, but these are very much *open* questions. Economic geography’s methodological culture tends to favor the oblique, the elliptical, and contrarian over the linear and incremental, and continuing chop and churn rather than cumulative consolidation. It is in this sense quite liberal and forgiving, if not in some respects informal (cf. Barnes et al., 2007). Methodological norms are quite elastic. Not much is codified; there are few rules, not even many guidelines. And yet at the same time, there can be said to be, de facto, a methodological culture, one that can and does change, and one about which it is surely healthy to be both self-aware and reflexive.

So where to start? Almost exactly 40 years ago, Doreen Massey and Richard Meegan convened an invitational workshop for industrial geographers (as they were then known) at the Open University. Where *and how* to start were among the questions that were debated back then (in the midst of “deindustrialization,” and the recognition of so-called regional problems, old and new). The workshop addressed questions of method, politics, and theorizing, all at the same time. What difference did it make (pro)actively to problematize the specifically capitalist nature of production, “to conceptualize it explicitly,” as the new generation of critical and more qualitatively-inclined, “intensive” researchers were inclined to do, as opposed to “tak[ing] the capitalist system as given [and then] exploring causal relationships within it,” in the tradition of more positivist, “extensive” models of inquiry (Massey and Meegan, 1985: 5; 2007: xi)? Refusing to accept “the system” as given meant that it was “necessary to conceptualize it as *capitalist*,” Massey and Meegan (1985: 6) argued; even when breaking off a piece (say, a local-scale case study of economic restructuring), “the parts of the system must still be conceptualized in terms of the specific nature of their social relations.” When tackling one of the “parts,” it was deemed necessary to take into account, and consequently to conceptualize and theorize, the constitutive relationship between that particular piece of the jigsaw and the moving puzzle that was the always-emergent “whole,” if only to be able to say what the case in question was a case *of*. For Massey and Meegan (1985: 5) this meant getting away from timidly empirical and self-limiting studies, it meant opening up the critical horizons by “break[ing] in at the level of the system as a whole.” But how?

Massey was fond of repeating the Althusserian line, “there is no point of departure” (quoted in Ijams et al., 1994: 107), which not only reflected antipathies to foundationalism and essentialism, but also commitments to open horizons of analysis, “enriching and complexifying” exploratory modes of explanation in ways that demanded the grounding and contextualization of what are sometimes called “general” theory claims.^1^ What she meant of course was there was no such thing as blank-slate, no *tabula rasa*, and no isolated site of historical origination, when the story could be said to begin at *t* = 0. Whenever and wherever one chose to start, or to “break in,” it could only be right in the thick of things, in mid flow, and also amid a tangle of deep interdependencies and articulated relations, causal tendencies and contingent conditions. Massey’s insistence on the distinctly “ungeological” interactions between successive rounds of investment (the geohistorical “combination of layers”) represented, in effect, her profoundly relational version of (or alternative to) path dependency, signaling how what goes before conditions and enables (but does not determine) what might be fashioned next, politically as well as “economically” (see Featherstone et al., 2013; Massey, 1995; Peck et al., 2018). Famously, this informed a take on the uniqueness of place, not as an inert ideographic category or bounded container of social processes, but as a site of relational interdependencies, emergent capacities, and generative interactions. From this perspective, there can be no place to start that is “typical” or “representative,” and no place to start that is neutral or random from either a political or a theoretical point of view. Instead, there is an obligation, not to say responsibility, to *engage with* (sociospatial) difference, something that Massey always insisted had to be more than a matter of nodding gestures, parenthetical asides or taking into account so-called contingencies, but “involves recognizing *from the start* the existence and importance of variety, and building that into the manner of initial conceptualisation” (Massey, 1995: 325, original emphasis).

Where we begin, in this sense, is inescapably tied up with *how* we begin, as well as how we think about (and theorize) beginning—whether we like it or not. The choices that we make, early on, about research design, problem definition, case-study specification, and the determination of fieldwork sites or spaces of inquiry, *cannot but* be tangled up with questions of theory and indeed politics. There is (and *can be*) no pretheoretical moment of methodological neutrality or inductive innocence, just as there is no way to suspend questions of politics and positionality. According to what criteria, then, is a methodologically responsible economic geographer to figure out where and how to start? There are no clear-cut or pregiven points of departure, no settled understandings of where stories “naturally” begin, and (therefore) no commonly accepted rules about where to start and how to “cut in,” on what is a moving *and uneven* terrain of relational connections and mutual interdependencies. Hence Massey’s injunction, “*from the start*” to address issues of conceptualization. In practice, economic geography’s methodological stock-in-trade, at least for the past few decades, has been to cut into problems mostly in the here-and-now, in the “restructuring present,” that is, tracking more-or-less contemporary processes of transformation, typically studied in real time, and usually in touch, concretely and socially, with the ground and indeed the action. But economic geographers, on the whole, tend to do so without explicit recourse to pregiven conceptual maps—be these of the contemporary world system, the extant geopolitical order, varieties of capitalism, or a hierarchy of (lagging/advanced) regions or (global) cities—being inclined, on the contrary, to be rather skeptical of such things, if not actively antagonistic to their pregiven contours and categories.

Fair enough. There is certainly nothing wrong with starting off with a critical disposition; on the contrary. And few are interested in the kind of rigid theories that seem to come with prefigured or foreclosed answers. But to set off in the apparent absence of a preliminary map, a rudimentary compass, or a worked-out sense of (theoretical) direction is a rather different matter. This is not to say, to be clear, that economic geographers are in the habit of launching their projects in states of theoretical ignorance. Clearly not. They spend a lot of time thinking about theory and theories, hold sometimes strong opinions about them, but usually prefer not to be tied down by them. Predispositions to critical reflexivity, various degrees of healthy skepticism . . . these can and should be considered to be disciplinary assets. But in relation to the matter at hand here, economic geographers tend to devote relatively little sustained attention to the theorization of research designs and case-study specification, or to the principled determination of points and places of analytical (and practical) departure. Inattention to this “pre-methodological moment” is perhaps surprising, given the significance that is generally attached to place, positionality, and perspective. After all, economic geographers are usually among the first to insist that uneven spatial development is an endemic feature of the capitalist (and more-than-capitalist) world, even as the appetite in the field for systemic, generalized, “structural” or otherwise mechanically principled theories of uneven development tends to be limited. Uneven spatial development represents an elemental condition of existence, of course, for the kind of local and regional studies that are conventionally conducted by economic geographers, but the explanatory presence, salience, and purchase of uneven spatial development *itself* will often, as a matter of explanatory practice, be rather nebulous—more background scenery than active problematic. There are, in truth, no abracadabra solutions to this conundrum, especially given the widespread tendency in the field to favor—productively, and for good reason—methods of inquiry and modes of explanation that are context-rich, experience-near, and socially eventful.

Two preliminary conclusions arise from the discussion so far. First, when it comes to the selection of research sites and case-study locations, there are no neutral, self-evident or typical places to start; nowhere is “average,” every site is itself situated; in a world of entrenched if not systematic uneven development, sticking a pin in the map will not do. Second, the question of starting points and places really has to entail some form of “initial conceptualization,” some form of proto-theorization and conceptual mapping. It follows that cases are *made* rather than simply being found. And consequently, places to start and domains of investigation are likewise coproduced with “starting” theories, initial conceptualizations, hunches and hypotheses, positions and perspectives, rather than simply being just out there. Placing and casing are both bound up with that issue of “initial conceptualization” too. Or to put it another way, they are coproduced with different “theory-method packages,” to invoke a formulation that circulates mostly outside economic geography with not much currency within (see Gehman et al., 2018; Silvast and Virtanen, 2023; Tavory and Timmermans, 2009). This begs the question of the kinds of theory-method packages, with what implications for casing and research design, might be identified within (and for) economic geography? There is certainly no singular or definitive answer to this question, but Table 1 summarizes a preliminary thought experiment that seeks to capture some of the diversity of explanatory cultures in economic geography. The purpose here is illustrative, to underscore the point that there are numerous ways as well as places to start. (Yet the very fact that economic geography does not have ready recourse to such things is itself telling.) So the purpose of the table is not to classify, less still to corral, discussions around different theory-method packaging styles, but rather to summon a vocabulary, and to invite a dialog, about how different explanatory orientations, casing strategies, and modes of theorizing might be imagined and “packaged,” in what combinations and permutations, to what ends.

**Table 1.** Explanations in economic geography: diverse approaches to theory-case packaging.

*Explanatory disposition*	Critical, strategic, stress-testing	Conjunctural, searching, reflexive	Corroborative, confirmative	Purposive, pragmatic	Synthetic, schematic, parsimonious
*Casing strategies*	Extended cases	Critical cases, crisis situations	Illustrative, affirmative, and comparative cases	Paradigmatic, heuristic cases	Ideal-type cases, sans contingency
*Theoretical purpose*	Reconstruction; remaking favored theories	Rearticulation; midlevel, generative theorizing	Application, exemplification, elaboration	Enhancement, extension, enrichment	Sharpening, refinement
*Theory-culture*	Oblique, (self) critical	Open, revelatory, radical	Validatory, curious, modest	Positive, (story)telling, consolidating	Essentializing, stylizing

There are many potential pathways through such “packaging” questions, not one. For my own part, I have been inclined to explore possibilities on the lefthand side of Table 1, recognizing that this is not for everybody. With this in mind, I conclude this brief intervention, while sensing that the discussion itself has only just started, by floating three propositions—all of them keyed into the matter of starting points and places. The first of these concerns the conventionally silenced C that is *combination*, the missing middle of the concept of uneven and combined development. The second proposition, a neglected C, concerns the often-overlooked questions of *casing*, case selection, and case-study design, questions that ought really to be quite fundamental for a field seriously concerned with the concrete facticity and commonsense reality of uneven geographical development. And the third proposition concerns an elusive C, the weakly codified practice of *conjunctural* analysis, recognized as a somewhat latent current in economic geography, but perhaps an emerging locus of methodological potential. Within the constraints of this short intervention, there is not the space for an extended elaboration. Instead, the following discussion hopefully indicates some places—in principle and practice—with which to start.

First, the silent C that is combination. Somewhat curiously, in retrospect, “uneven development” in its received, contracted form, tends to elide (conceptually as well as semantically) the process of *combination* that represented the relational heart of the original concept of uneven and combined development (Peck, 2019). More than a century ago, the idea of combined or recombinant development emerged out of efforts to transcend the limitations of teleology and stage-modeling, subsequently providing a warrant for exploring those variegated conditions of (super)emergence, within and between places, through which accumulation projects and developmental pathways are concatenated and hybridized.^2^ Understood as an in situ and geographically sited process, this resonates with Massey’s relational treatment of the “combination of layers.” This was never simply a matter of the successive sedimentation, historically, of industrial practices and work cultures, as if one layer superseded and obliterated its predecessor. Instead, it signaled the complex and continuing *interaction* between layers, a geographically specific form of mutual determination that “really does mean combination, with each side of the process affecting the other” (Massey, 1995: 313; 1984: 117–120). The notion of the spatial division of labor, furthermore, underscores the *interregional* interdependencies that are successively remade through this multisided process, one that entails open-system complexity, lots of it, although not unpatterned indeterminacy per se. In terms of potential places to start, criteria relating to the coexistence, combination, and variable articulation of industries, technologies, labor processes, modes of social reproduction are suggested here.

Yet if the basic principles of such processes of relational combination were established in Massey’s project, its unfinished business includes a raft of methodological implications and opportunities. To problematize combination (as a corollary of uneven development; as its dialectical companion, or neglected sibling) is to engage the ontological premise of “more-than-one” (cf. Rosenberg, 2006: 318), to think with heterogeneity and difference from the get go. Multisite comparisons of different kinds have an obvious role to play here, but there is also a prompt for single-site investigations seriously and explicitly to problematize *more than one thing*—and to build this sense of heterogeneous coexistence, multicausality, and intersectionality “into the manner of initial conceptualization.” Cross-border regions of different kinds certainly facilitate (not to say require) moves such as this, as do relationally-comparative research designs predicated not on the side-by-side comparison of nominally discrete cases but on the relational coexistence of differently articulated, heterogeneous configurations (for instance, various combinations of production systems or labor processes on the one hand, and governance regimes or patterns of social reproduction on the other). Innovative approaches to multisite comparison have especially generative roles to play here (see Hart, 2018; Leitner and Sheppard, 2020), terrain that really ought to be much more familiar to economic geographers than in practice it appears to be (cf. Peck, 2012).

This is where the neglected C of case-study specification and rationalization comes in. If “casing” is a theoretical problem, it is also presents opportunities for theoretical development and reconstruction. Burawoy’s (1998) take on the extended case approach comes with rich, if mostly implicit, geographical mandate: to begin with those places that present opportunities to stress-test and problematize our working theories, to *set out to* reconstruct rather than simply apply them. Apparently anomalous situations, unusual configurations, and ostensibly contradictory combinations might all figure here, but note that they figure *in relation to* theoretically framed expectations or initial conceptualizations. There are rationales for getting started here, although there is a need to guard against reflexive recourse to what Massey (1995: 318) called “taxonomic disaggregation,” the habit of breaking down objects and domains of inquiry into smaller (and, on the face of it, more internally homogeneous) pieces, and concentrating “close up” on one-sided characteristics of those cases (say, as centers of innovation or sites of industrial reinvention). This kind of methodological localism, with its tacit privileging of *internal* conditions and causes, is quite commonplace in economic geography, where there are long-established (and often well-founded) suspicions about the smothering effects associated with big-picture or top-down theories, and with the flattening consequences of universalist explanation, convergence claims, and equilibrium models. Instead, depictions of locally-scaled variety are frequently mobilized in the service of modifications or corrections to some allegedly general rule or overblown claim; a vivid portrayal of locally-scaled *exception* may even disprove that general rule or overblown claim. But what if, rather than atomizing local difference through “disaggregation” and turns toward the microanalytic (cf. Cochrane, 1987), there were moves instead purposefully to *extend* cases and frames of analysis (cf. Burawoy, 1998; Hart, 2018), so as to engage difference, disjuncture, combination, and coexistence across more expansive terrains?

At least two possibilities for case-extending moves, transcending methodological localism, suggest themselves (once again with the proviso that these are particular strategies among many). In one approach, the boundaries of cases are permeated, and the spaces between them populated, by setting out to explore *field*s constituted through connected practices or processes—a methodological strategy that has been used in the policy mobilities literature (see e.g. Peck and Theodore, 2015). Casing here is not really about bounding, or the capture of some internal essence, or the privileging of particular sites; instead, it is about relations and connections, and tracking between and across multiple sites. Process tracing and pattern recognition have significant roles here, working across sites not to generalize, synthesize, or find common denominators, but to engage uneven spatial development itself.

A different kind of move beyond methodological localism entails the embrace of scale. More than just thinking big, this can be a warrant for “macroeconomic” geographies that do not simply substitute large categories of analysis (or big cases) for smaller ones, but which instead endeavor to gather uneven-and-combined development into their analytical gaze, reach, and ambit. Rather than uneven geographical development representing a thinly theorized hinterland, positioned in the background of locally-scaled investigations, there would instead be a critical orientation to problem spaces, situations of concern, moments of crisis or accelerated transformation, inflection points, and places to start that *take in and take on* questions of sociospatial difference, compound causality, recombinant development, contradictory conjunction, and variable (dis)articulation. Instead of taking the uneven development of variegated capitalism as a given or precondition, *within which* cases are then located, what if cases are instead selected so as to perturb and disturb the (presumed) contours of variegation itself? This is a remit for starting with moments of crisis, conflict zones, fault lines, disputed territories, and sites of systemic frailty or fracture (see Peck, 2023a).

These are the kinds of unsettled sites and discomfort zones to which conjunctural analyses are especially well suited, understood as a distinctive approach and methodological ethos (see Clarke, 2018; Leitner and Sheppard, 2020; Pickles, 2012). Characteristically unbounded, conjunctural analyses will often begin with (or set out from) the localized here-and-now—economic geography’s home turf, if you will—but their relational and case-extending epistemology calls for a series of next steps that characteristically spiral out, in a searching manner, to situate, and to historicize, in order to “radically” contextualize (Grossberg, 2019). The resulting “thickly theorized” cases tend to say more about conflict and contradiction than coherence or unity, speaking more to multiple, intersecting processes of conditioning and causality than to the play of one-sided abstractions or the refinement of ideal-typical configurations.

Conjuncturalism has been invoked here as an elusive C because, while it may have been a nascent or incipient presence in economic geography at least since the time of Massey’s (1984) decisive intervention, the approach (*qua* specified approach) has never really been sustained, and certainly not codified. This is understandable, because conjunctural analysis, as a mode of critical practice, really requires practice (and often collaborative practice too), while also being pretty much antithetical to procedural formalization. Accustomed as it has become to improvising and experimenting, to relatively “open” modes of theorizing, and to making paths more by walking rather than by following predefined rules, economic geography might even possess some methodological advantages on this score—working what Stuart Hall once called the “stony ground” of conjunctural analysis. Where this might lead is an open question, but at least it would be a place to start.

## Notes

1. Massey maintained that “the aim must be to *use* general theory not to reject it. But to use it means to use it in specific contexts—for there are no ‘general’ ones, in that sense, to use it on” (Massey, 1989: 695, original emphasis). On some of the implications of this approach to conjunctural theorizing and “radical contextualism,” see Pickles (2012), Grossberg (2013, 2019), and Peck (2023b).

2. What Trotsky famously dubbed the “privilege of historic backwardness” afforded notionally lagging regions the option (if not the imperative) to “skip” moments in the historical developmental paths experienced by other regions (those that today might be labeled, rather problematically, “advanced”), yielding a “peculiar combination of different stages in the historic process” through which the process of development “as a whole acquires a planless, complex, combined character” (Trotsky, 2008 [1932]: 4). This germinal conception of recombinant development was read as an “*amalgam* of archaic with more contemporary forms” (Trotsky, 1976 [1938]: 583, emphasis added).
